# Introducing the first link of the Chain of Survival to preschool children: a feasibility observation

**DOI:** 10.1016/j.resplu.2026.101456

**Published:** 2026-08-19

**Authors:** Burak Çelik, Funda Yusufoğlu, Kadir Emre Yakışık, Nilgün Göleş

**Affiliations:** a Department of Emergency Medicine, Fatih Sultan Mehmet Training and Research Hospital, University of Health Sciences, Istanbul, Türkiye; b Kopuzlar Foundation Preschool, Ministry of National Education, Istanbul, Türkiye

To the Editor,

Children can learn to recognise an emergency and call for help long before they can perform CPR. This is the first link of the Chain of Survival and a core aim of the KIDS SAVE LIVES initiative.^1^, ^2^ Even so, there is little evidence on whether it can be taught this early. We conducted a single, play-based session with one class of 5-year-old children (*n* = 25) as part of a district social responsibility project, and report our procedures and observations using the TIDieR and GREET frameworks.^3^, ^4^

The session was built around a toy bear about 50 cm tall. We first discussed unsafe situations using simple drawings. We then placed the bear, out of the child’s sight, at one of several set points: near cables, next to a stove, next to a tea urn, on a high table, or in an open space. Each child decided whether the spot was safe or dangerous. In dangerous settings, the child called out to the bear from a safe distance rather than approaching it; in safe settings the child knelt down, gently checked the bear, and practised look, listen and feel. The children then sought help from an adult and made a pretend emergency call on a toy phone. We showed the recovery position and let the children try chest compressions on the bear to convey the rhythm, without aiming for a full cycle; we stated clearly that real chest compressions are an adult responsibility and that the child’s role is to stay safe and get help. Every spot marked as risky was cleared of any real danger beforehand. The session ended with a visit to a 112 ambulance.

Eighteen of the 25 children (72%) took part in the active steps (Table 1); participation remained voluntary, and the children who preferred to observe were not pressured. Among those who took part, we observed children distinguish safe from hazardous settings, seek help from an adult, and complete the toy-phone call, although these steps were not formally scored. When asked for an address, only 3 of the 25 could give their home address; the remaining active participants (15/18, 83%) completed the task by giving the school name. This is in line with earlier reports that young children find it hard to convey their location,^5^ and suggests that teaching the school name may be a practical, realistic target for this age group.

**Table 1:** Intervention components and observations (single session; 25 children enrolled).

**Parameter**	**Result**
Active participation	18/25 (72%)
Observation-only preference	7/25 (28%)
Distinguished safe vs. hazardous settings	Completed by participating children (observed; not formally scored)
Demonstrated adult help-seeking	Completed by participating children (observed; not formally scored)
Completed toy-phone emergency simulation	Completed by participating children (observed; not formally scored)
Stated own home address (all enrolled)	3/25 (12%)
Gave school name instead (active participants)	15/18 (83%)

Values come from observational notes taken during the session; no validated quantitative outcome measure was used.

A short, teacher-supervised, play-based session appears feasible and engaging for introducing these first steps: stay safe, get help, and make a call. The study is small, took place in one class on a single day, and used no formal test of what the children learned; the findings are therefore observational rather than conclusive, and need to be confirmed with structured pre- and post-intervention measurement in larger, controlled studies. As a school activity rather than a clinical study, ethics-committee approval was not required under national regulations; appropriate institutional permissions and written parental consent were obtained.

## Author roles

**Funda Yusufoğlu:** Preschool Teacher, Kopuzlar Foundation Preschool.

**Nilgün Göleş:** Head Teacher (Başöğretmen), Kopuzlar Foundation Preschool, Ministry of National Education.

## CRediT authorship contribution statement

**Burak Çelik:** Conceptualization, Data curation, Methodology, Resources, Supervision, Writing – original draft, Writing – review & editing. **Funda Yusufoğlu:** Conceptualization, Methodology, Resources. **Kadir Emre Yakışık:** Conceptualization, Investigation, Writing – original draft, Writing – review & editing. **Nilgün Göleş:** Methodology, Resources.

## Funding

None.

## Declaration of competing interest

None declared.
